# A Comprehensive Methodology for Optimizing Read-Out Timing and Reference DAC Offset in High Frame Rate Image Sensing Systems

**DOI:** 10.3390/s23167048

**Published:** 2023-08-09

**Authors:** Jaehoon Jun

**Affiliations:** Department of Electrical Engineering, Inha University, Incheon 22212, Republic of Korea; jaehoon.jun@inha.ac.kr

**Keywords:** ADC decision timing, high frame rate, high resolution, image sensor, power efficiency, ramp reference offset, read-out timing optimization

## Abstract

This paper presents a comprehensive timing optimization methodology for power-efficient high-resolution image sensors with column-parallel single-slope analog-to-digital converters (ADCs). The aim of the method is to optimize the read-out timing for each period in the image sensor’s operation, while considering various factors such as ADC decision time, slew rate, and settling time. By adjusting the ramp reference offset and optimizing the amplifier bandwidth of the comparator, the proposed methodology minimizes the power consumption of the amplifier array, which is one of the most power-hungry circuits in the system, while maintaining a small color linearity error and ensuring optimal performance. To demonstrate the effectiveness of the proposed method, a power-efficient 108 MP 3-D stacked CMOS image sensor with a 10-bit column-parallel single-slope ADC array was implemented and verified. The image sensor achieved a random noise of 1.4 e^-^rms, a column fixed-pattern noise of 66 ppm at an analog gain of 16, and a remarkable figure-of-merit (FoM) of 0.71 e^-^·nJ. The sensor utilized a one-row read-out time of 6.9 µs, an amplifier bandwidth of 1.1 MHz, and a reference digital-to-analog converter (DAC) offset of 512 LSB. This timing optimization methodology enhances energy efficiency in high-resolution image sensors, enabling higher frame rates and improved system performance. It could be adapted for various imaging applications requiring optimized performance and reduced power consumption, making it a valuable tool for designers aiming to achieve optimal performance in power-sensitive applications.

## 1. Introduction

Various types of sensing systems are used in the era of the Internet of Everything [1,2,3,4,5,6,7,8,9,10]. With the advent of the Fourth Industrial Revolution, the demand for image sensor-related products has been increasing rapidly [5,6,7,8,9,10]. This enormous demand is not limited to consumer products and is continually expanding into defense, security, privacy, autonomous driving, and space science. 

As the applications of image sensor systems become more diverse, they require extreme characteristics that are difficult to achieve, such as 200-megapixel (MP) resolution, 140 dB dynamic range, ultra-compact multi-functionality, and invisible ray cameras [11,12,13]. Additionally, increased power consumption and heat generation are issues as more image functions are required for high-resolution cameras, such as fast auto-focus (<0.3 s) and slow-motion video with ultra-high frame rates (>240 frames/s) [13,14,15,16]. Moreover, there has been a recent demand for ultra-low-power characteristics for always-on-display capabilities in imaging systems.

There are many ways to read out the output of a pixel array, but in most cases, an array of thousands of analog-to-digital converters (ADCs) is integrated into a column-parallel architecture and used to digitize the pixel output. A single high-precision ADC must be implemented with a sub-micron pitch (<1 μm) to realize a high-resolution image sensing system with low-noise characteristics. Therefore, single-slope ADCs with relatively simple structures are commonly used as pixel digitizers. 

When utilizing a column-parallel ADC array to digitize the output of a pixel array, it is crucial to cancel out the dark noise of the pixels to obtain a high-quality image. To suppress the low-frequency noise of a pixel, most state-of-the-art image sensing systems use the digital correlated-double sampling (CDS) technique, which subtracts two digitized outputs of a pixel before and after it receives external light [17,18]. The digital CDS technique requires twice as many ADC operations, making the system timing budget insufficient for modern image systems with high resolution and a high frame rate. Furthermore, there are many other complex considerations for read-out timing, such as auto-zeroing (AZ), analog CDS, pixel reset, and the shutter. 

This paper proposes a read-out timing optimization methodology utilizing an optimal reference offset for high-resolution, high-frame-rate image sensing systems. It includes considerations for the pixel array, the digital-to-analog converter (DAC) for the ramp reference of a single-slope ADC, and both analog and digital CDS techniques. With this timing optimization methodology, the amplifier bandwidth of the power-hungry comparator array can also be optimized, enabling energy-efficient image sensing. The rest of this article is organized as follows: Section 2 describes the architecture of modern image sensing systems. The proposed read-out timing optimization methodology is discussed in Section 3. Section 4 presents an implementation example with the proposed timing optimization. This paper concludes in Section 5.

## 2. Image Sensor Architecture

**Figure 1:**
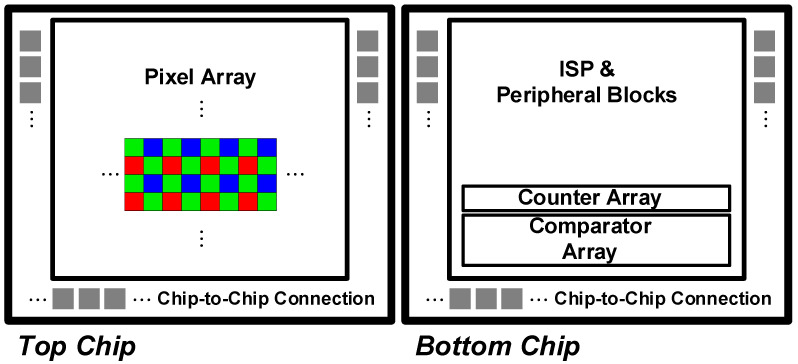
3-D-stacked image sensor architecture.

An imaging system has an inevitable trade-off between system performance and power consumption. To optimize this complex timing budget, the first step is to thoroughly understand how advanced image sensors are configured. Image sensors have evolved to implement pixel arrays and digitizer arrays on separate chips in stacks of three-dimensional (3-D) integrated circuits (ICs) using through-silicon via (TSV) or Cu-Cu connection techniques to achieve a small form factor [7], as shown in Figure 1. With the 3-D stacked architecture, an upper chip for the pixel array and a lower chip for the digitizer array can be separately implemented using optimal process technologies. Therefore, the rest of this section describes the structure of pixel and digitizer arrays for read-out timing analysis and system optimization.

### 2.1. Pixel Structure

**Figure 2:**
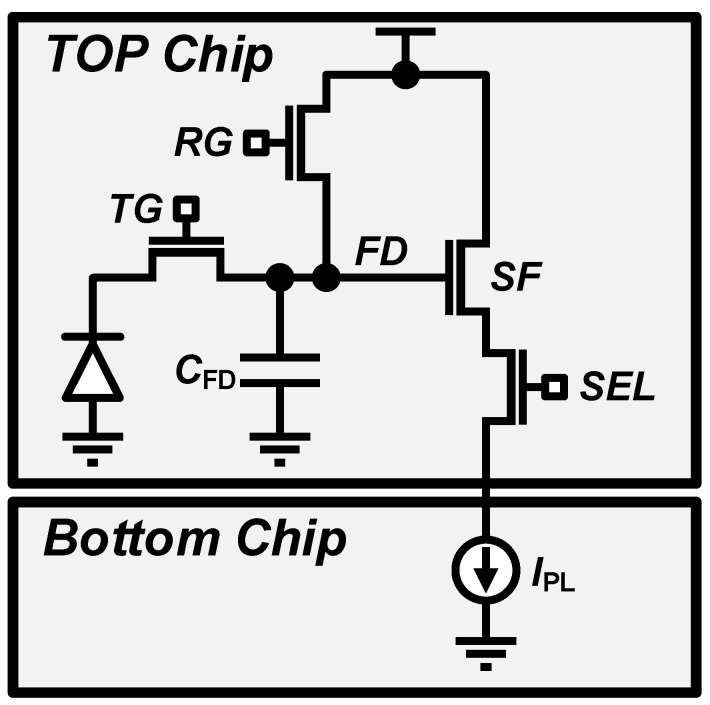
Simplified 4-T active pixel structure.

Figure 2 shows a simplified active pixel sensor (APS) structure with one pinned photodiode and four transistors (4-T) for a CMOS image sensor (CIS) [19,20]. A photodiode in a pixel acts as a light-to-electron converter. When incident light is applied, a photodiode in the pixel produces electrons proportional to the intensity of the light. The four MOS transistors consist of a row selection transistor (SEL), a pixel reset gate (RG), a charge transfer gate (TG), and a source follower (SF) buffer. The output of the pixels is read out row-by-row with the rolling shutter method, so the SEL transistor is used to select the pixel row to digitize. After row selection, a reset sequence is required to eliminate residual electrons by turning on the RG before using the pixel as a sensor. 

Once the pixel reset is completed, electrons are generated by the photodiode receiving incident light and transferred to a floating diffusion (FD) node by turning on the TG. An FD node has a capacitance on the order of fF or smaller, and electron-to-voltage conversion with a conversion gain (CG) occurs during this photodiode-to-FD charge transfer process. Furthermore, the FD node voltage becomes the output voltage of the pixel through the in-pixel SF buffer, which is digitized by the following ADC.

**Figure 3:**
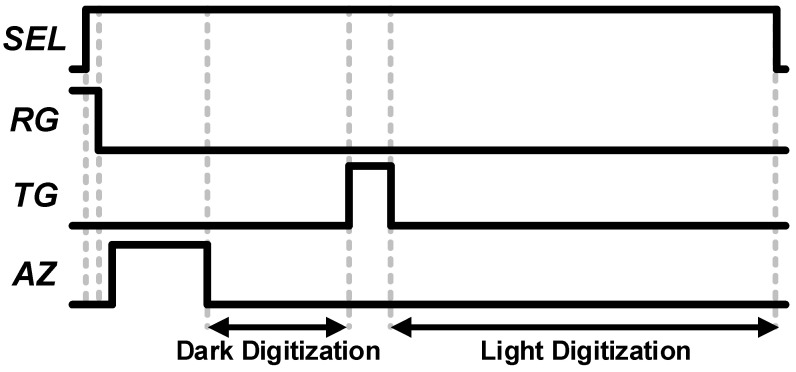
Simplified timing diagram of the pixel with the digital CDS technique.

Figure 3 shows the timing diagram of the 4-T pixel with a digital CDS technique. To cancel out the pixel output variation, including pixel reset noise, a digital CDS technique is widely used. For a digital CDS function, two digitizations are performed, and the digital difference is equivalent to the perceived intensity of the light. Therefore, the dark signal is read before the TG is turned on, and the light signal is read after the TG is turned on.

### 2.2. Read-Out IC Structure

**Figure 4:**
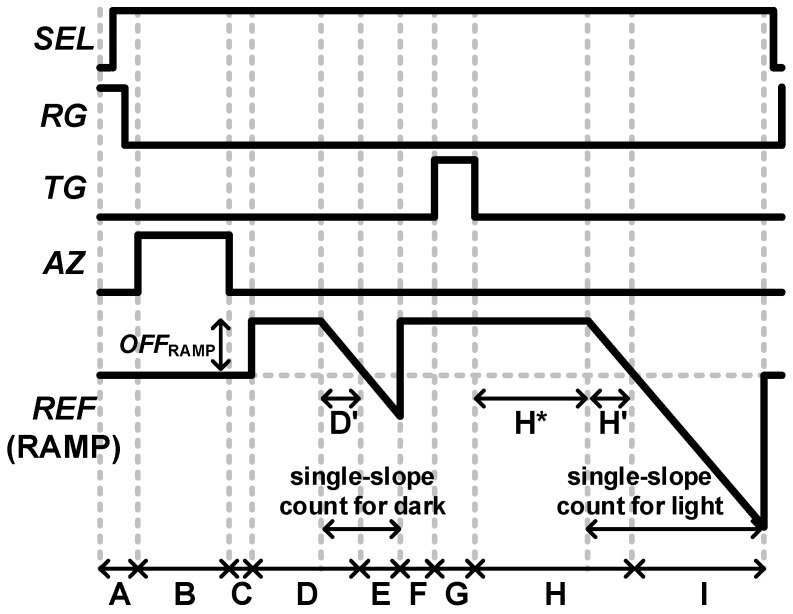
Timing diagram of the image sensor with the digital CDS technique.

The SF elements in the pixel array on the top chip require a current load (*I*_PL_) to function properly, which is usually implemented on the bottom chip. As shown in Figure 4, once the pixel reset (period A) is complete, the auto-zero (AZ) operation (period B) of the ADC can be started. During the AZ phase, DC offset and flicker noise are stored for the analog CDS operation, and a self-bias network is operated to determine the operating bias of the amplifier. When the AZ operation is completed, the digitizer reads out the data before and after receiving the incident light and then finds the difference to obtain the result of the digital CDS (periods C to I). In the single-slope counting sections (periods E and I), a comparator compares the pixel output with the reference voltage, which is the ramp signal implemented based on the DAC. Additionally, the reference offset (*OFF*_RAMP_) can be added before the start of the ramping to prevent missing the dark signal, and the added offset is naturally canceled out with the digital CDS technique.

## 3. Read-Out Timing Optimization Methodology

**Figure 5:**
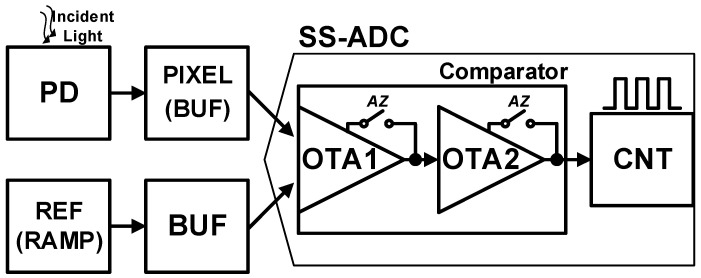
Simplified block diagram of the image sensor.

A simplified block diagram of an image sensor is shown in Figure 5. To achieve a column-parallel ADC architecture, a comparator must be composed of a simple structure, which is a 5-transistor first amplifier and a common-source second amplifier. The two-stage amplifier with an open-loop topology is well-used in an ADC array structure [7,8,18,21]. The read-out sequence of the image sensor from period A to I (one-row read-out time) is repeated until the entire pixel array has been read row by row. Therefore, the one-row read-out time can be determined based on the pixel resolution and the target frame rate of the image sensing system. For example, if a 100 MP image sensor (10,000 × 10,000) is to be digitized at a target of 10 fps, the one-row read-out time would be 10 μs in a single ADC per single pixel column (1 ADC/col) structure. The one-row read-out time should be carefully distributed from period A to period I without any redundant or bottleneck periods. In this paper, an advanced read-out timing optimization methodology is proposed with an optimum reference offset.

### 3.1. Period A: Reset

At the beginning of every horizontal read-out time, a pixel row for digitization should be selected using the SEL transistor. Additionally, a reset operation at the FD node should be completed to empty the FD capacitor *C*_FD_. The *k*T/C noise generated during the reset period is suppressed by the digital CDS technique. This reset and selection of the pixel are relatively independent of the image resolution and can be defined as an absolute time interval according to a pixel structure.

### 3.2. Periods B and C: AZ

Before starting the AZ operation, the RG is turned off, which causes a voltage fluctuation (Δ*RG*_OFF_) that is transferred to the bottom digitizer chip through the 3-D chip-to-chip connection. During the AZ period, therefore, the effect of the voltage fluctuation from the top pixel chip should be sufficiently settled, as should the operation of the amplifier to determine the DC bias and store low-frequency noise. In this period, the amplifier of the single-slope ADC is in a very fast unity-gain configuration and has a very small time constant. Therefore, the settling bottleneck induced by the Δ*RG*_OFF_ is the output of the pixel, which is the input of the ADC. With the negligible time constant of the ADC, the resistance for the RC time constant is determined by the transconductance of the pixel source follower, the on-resistance of the pixel selection transistor, and the metal line resistance of the pixel output. The time constant and slew rate of the SF can be obtained as follows:

*τ*_SF_ = (1/*g*_m,SF_ + *R*_SEL_ + *R*_LINE_)·C_LINE_, (1)

*SR*_SF_ = *I*_PL_/*C*_LINE_ (2)

where *g*_m,SF_ is the transconductance of the SF, *R*_SEL_ is the on-resistance of the SEL, and *R*_LINE_ is the line resistance from the pixel to the ADC, including the chip-to-chip connection line. In the worst-case settling situation, slewing is caused by the condition *τ*_SF_·*SR*_SF_ < Δ*RG*_OFF_, and the required settling and slewing voltage can be given by:

Δ*V*_SETTLE,B_ = *τ*_SF_·*SR*_SF_, (3)

Δ*V*_SLEW,B_ = Δ*RG*_OFF_ − *τ*_SF_·*SR*_SF_. (4)

Then, the required time for the slewing and settling can be derived as follows:

*T*_SLEW,B_ = Δ*V*_SLEW,B_/*SR*_SF_, (5)

*T*_SETTLE,B_ = *τ*_SF_·*In*(Δ*V*_SETTLE,B_/*E*_TARG,B_) (6)

where *E*_TARG,B_ is the target achieved settling error in period B, and the timing budget for the period can be obtained as *T*_SLEW,B_ + *T*_SETTLE,B_.

For period C, the voltage fluctuation induced by the turn-off signal of the AZ is well suppressed by the pseudo-differential amplifier topology of the ADC. Therefore, the timing budget for this period can be defined as a small absolute value for non-overlapping clock timing.

### 3.3. Period D: Reference Offset and Its Counting

In the ideal case, the ADC decision time of the dark signal is the end of period D. However, the pixel output has a wide output variation, so it can be missed without the ramp offset (*OFF*_RAMP_). Therefore, in general, the dark ramping period should be long enough to include the output variation before digital CDS. However, if the ramp settling at the output of the amplifier is not sufficient until the time of the ADC decision, this settling error cannot be suppressed by the digital CDS technique. To achieve the high color linearity (CL) characteristic of an imager, the linearity relative to the ideal dark signal should be constant with respect to the light intensity. The color linearity (CL) error can be expressed as follows:

(7) $$\left(1-\frac{\left(O_{X}-O_{0}\right)/\left(O_{X,\mathrm{IDEAL}}-O_{0,\mathrm{IDEAL}}\right)}{\left(O_{\mathrm{REF}}-O_{0}\right)/\left(O_{\mathrm{REF},\mathrm{IDEAL}}-O_{0,\mathrm{IDEAL}}\right)}\right)\cdot 100$$

where *O*_X_ is the digitized output with the external incident light equivalent to the *X* LSB input, *O*_0_ is the output with no input, and *O*_REF_ is the output with the high code LSB input for the ratio calculation. In addition, *O*_X,IDEAL_, *O*_0,IDEAL_, and *O*_REF,IDEAL_ represent the ideal output values without any settling error. In high-resolution image sensors, the remaining settling error can thus degrade the CL error.

In this paper, a read-out timing optimization methodology is proposed to find the optimal reference DAC offset with the optimal settling error. There are two factors that contribute to settling errors in this period. The first factor is the voltage fluctuation due to *OFF*_RAMP_. With the reference offset and the target achieved settling error in period D (*E*_TARG,D_), the timing budget for this period is given by:

(8) $$\tau_{\mathrm{OTA}1}\;\ln\left(OFF_{\mathrm{RAMP}}/E_{\mathrm{TARG},D}\right)$$

where *τ*_OTA1_ is the time constant of the first amplifier, which is directly related to the bandwidth of the amplifier and can be approximated by the time constant of the single-slope ADC (*τ*_ADC_).

**Figure 6:**
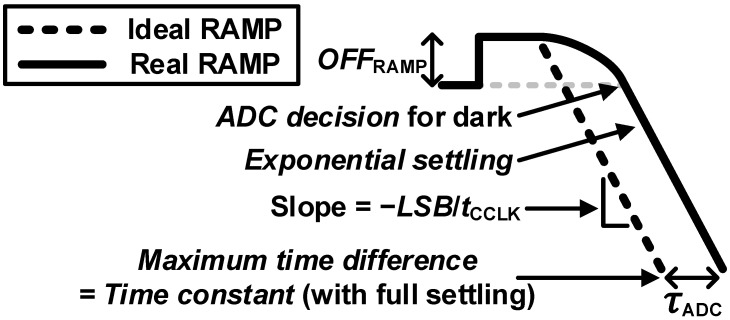
Ideal and realistic reference ramp waveforms.

The second factor that affects settling error in this period is ramp settling. Figure 6 shows ideal and realistic reference ramp waveforms, where *t*_CCLK_ is the unit-time step of the counter clock frequency. The reference ramp signal with a finite amplifier bandwidth of the following ADC causes a time-variable delay at the output of the ADC. At the start of the ramp, this time-variable delay is zero, which is the minimum delay. After sufficient settling time, the ramp delay gradually increases to the time constant of the amplifier, which is the maximum delay. In a single-slope ADC, the decision time is directly digitized by a following counter; thus, this ramp settling error must be well suppressed before the ADC decision.

For an ideal ramp, the time to count *OFF*_RAMP_ with an input signal of *X* LSB is given by:

(9) $$\left(\frac{OFF_{\mathrm{RAMP}}\;\;}{LSB}+X\;\right)\cdot t_{\mathrm{CCLK}}.$$

For a realistic ramp, however, the time taken for a decision can be determined by finding the zero-crossing solution of the following equation:

(10) $$\frac{-LSB}{t_{\mathrm{CCLK}}}\cdot \left(t-\tau_{\mathrm{OTA}1}\cdot (1-e^{\frac{-t}{\tau_{\mathrm{OTA}1}}})\right)+X+OFF_{\mathrm{RAMP}}.$$

Using (9) and (10), the CL in (7) can be estimated with an *X* LSB input and a reference input.

**Figure 7:**
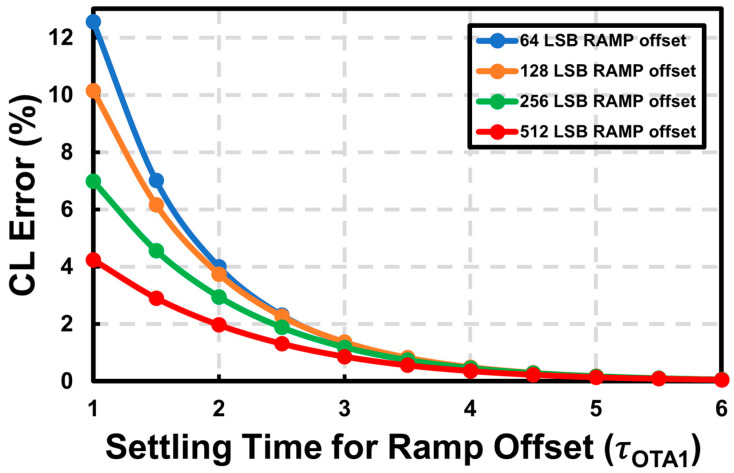
Calculated CL error versus settling time for *OFF*_RAMP_.

Figure 7 shows the CL error estimation versus the settling time budget for *OFF*_RAMP_ with an input of 10 LSBs and a reference of 256 LSBs. As an example, to ensure linearity characteristics above 99%, the minimum time for period D′ can be chosen as a relative value of 3τ_OTA1_. As shown in Figure 7, with a sufficient settling time of more than 4τ_OTA1_, the CL error becomes relatively independent of the ramp offset.

### 3.4. Period E: Dark Counting

Although the ideal decision timing of the dark signal is at the end of period D, the ramping period should be longer to include the peak-to-peak variation of the pixel output. If the ramping period is too short to include all pixel output, the fixed-pattern noise (FPN) of the output image is severely degraded. Therefore, it is important to budget the ramping period to the appropriate time, which can be iteratively determined between Monte-Carlo simulation of the ADC and the timing optimization method presented in this paper. After the iterations, the timing budget for period E can be defined.

### 3.5. Periods F, G, and H: TG

After the reset counting, the TG of the pixel must be turned on to transfer the electrons accumulated at the PN junction of the photodiode to the FD node for light counting. After reset counting, a small timing margin before turning on TG is required to avoid clock overlapping, so allocating a small absolute time is enough for period F.

For period G, the on-time of the TG should be long enough to allow sufficient photodiode-to-FD charge transfer. By comprehensively considering the structure and process of the pixel array, including back deep trench isolation (BDTI)/front deep trench isolation (FDTI), and front-side illumination (FSI)/back-side illumination (BSI), the timing budget for the on-time of the TG can be defined, which is independent of the ADC.

When the TG is turned off, a voltage fluctuation (Δ*TG*_OFF_), which is similar to Δ*RG*_OFF_ in period B, is induced and transferred to the digitizer chip. Since the ramping time in period Hʹ is the same as that in period Dʹ, which is used for *OFF*_RAMP_ ramping, the settling time for Δ*TG*_OFF_ can be optimized with period H*. At the pixel output, the required settling and slewing voltages can be derived as follows:

Δ*V*_SETTLE,H_ = *τ*_SF_·*SR*_SF_, (11)

Δ*V*_SLEW,H_ = Δ*TG*_OFF_ − *τ*_SF_·*SR*_SF_. (12)

The minimum time budgets for the slewing and settling voltages are then given by:

*T*_SLEW,H_ = Δ*V*_SLEW,H_/*SR*_SF_, (13)

*T*_SETTLE,H_ = ln(Δ*V*_SETTLE,H_/*E*_TARG,H_) (14)

where *E*_TARG,H_ is the target achieved settling error in period H, and the minimum time budget for period H* can be calculated by:

(*T*_SLEW,H_ + *T*_SETTLE,H_) − *T*_D′_ (15)

where *T*_D′_ is a chosen time budget for period Dʹ, considering the result shown in Figure 7.

### 3.6. Period I: Light Counting

For the single-slope counting of the light digitization, the ADC decision timing is dependent on the light intensity. If there is no light coming into the pixel chip, the ADC decision occurs with the same timing as the dark digitization. If there is detectable light, stronger light intensity leads to a later ADC decision. Therefore, the light counting period should sufficiently cover the pixel output range, and then the count of this period must be longer than 2^N^ LSB with an N-bit single-slope ADC. The timing budget for this period is then given by:

(2^N^ + *COUNT*_MARGIN_)/*t*_CCLK_ (16)

where *COUNT*_MARGIN_ is a single-slope counting margin that considers the system offset, mismatch, noise, and PVT variation.

### 3.7. Timing Optimization

Based on the timing analysis of each read-out period, an optimal timing diagram for a high-resolution image sensor can be derived. By utilizing the proposed timing optimization methodology with an optimal offset of a ramp reference, an optimized time for each period can be assigned, and an optimal ramp offset and amplifier bandwidth can also be achieved. 

**Figure 8:**
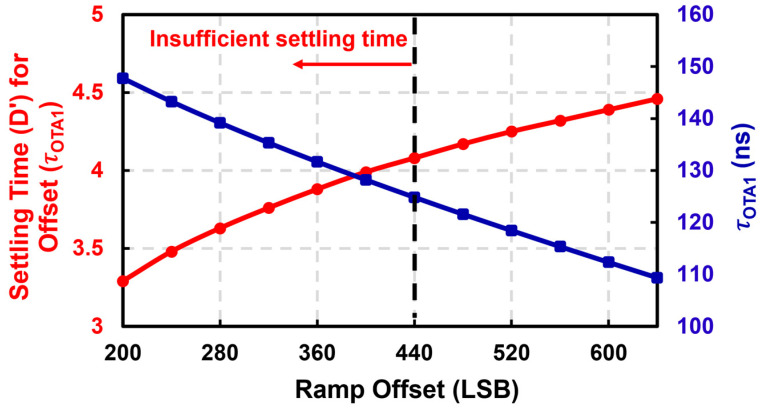
Estimated settling time for ramp offset and the time constant of the amplifier versus ramp offset.

For example, consider a 12,000 × 9000 pixel array that needs to be digitized with a 12-bit ADC array at 15 fps. The ADC array needs to process the pixel output of 9000 rows 15 times in 1 s, and a one-row read-out time is then 7.4 μs. With a specific pixel structure, system architecture design, and circuit simulation results, design parameters for a high-resolution image sensor can be achieved, as shown in Table 1. With the design parameters, the settling time for ramp offset and the time constant of the amplifier versus ramp offset can be calculated, as shown in Figure 8. A large ramp offset is required to ensure a sufficient ramp offset settling time, which in turn requires a small time constant, which increases power consumption.

Through the iterative calculation based on the other parameters in Table 1 and the equations in Section 3, optimized time budget results can be achieved, as shown in Table 2. With the proposed timing optimization methodology, an optimal reference offset of 440 LSB was achieved. Considering the effect of the PVT variation, a reference offset of 480 LSB can be chosen. Furthermore, an optimal amplifier time constant of 121.6 ns is also derived, which is equivalent to a bandwidth of 1.31 MHz. Without optimizing the reference offset as proposed in this paper, the power efficiency of an image sensing system becomes very poor. For example, an amplifier bandwidth of 2.49 MHz would be required to maintain the same CL error with an unoptimized reference offset of 256 LSB.

With this approach, the power consumption of the amplifier array, which is one of the most power-hungry circuits, can be minimized. This can increase the system’s energy efficiency or frame rate by minimizing one-row read-out timing.

## 4. Implementation and Experimental Results

**Figure 9:**
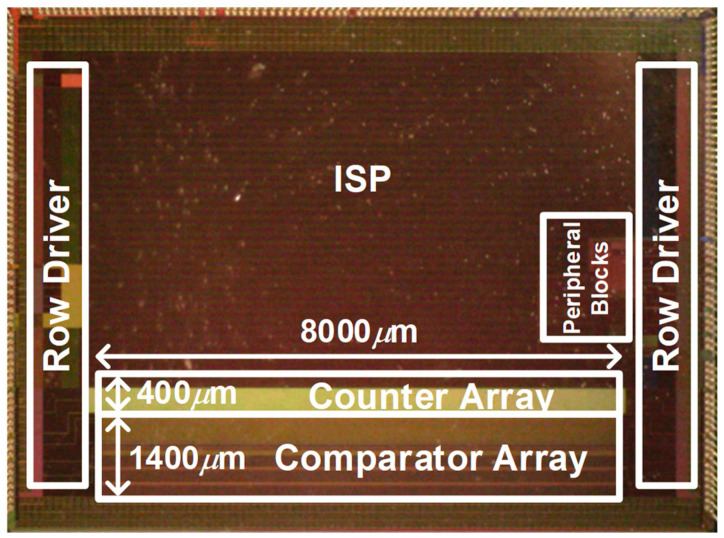
Microphotograph of the digitizer chip.

A power-efficient digitizer array for verifying the proposed time budgeting method is implemented in a 28-nanometer process with a chip size of 47 mm^2^. The prototype digitizer is designed with an optimal reference ramp offset and a 10-bit column-parallel single-slope ADC array. Figure 9 shows an annotated microphotograph of the digitizer chip, which can be stacked with a pixel chip. The width of the implemented ADC is only 1.005 μm, with a height of 1800 μm. The heights of the comparator array and counter array of the ADC are 1400 μm and 400 μm, respectively. The comparator array and counter array are operated with a supply voltage of 2.8 V and 1 V, respectively. The peripheral blocks include a DAC for reference ramp signal generation, a voltage doubler for the pixel chip, and reference current generation.

**Figure 10:**
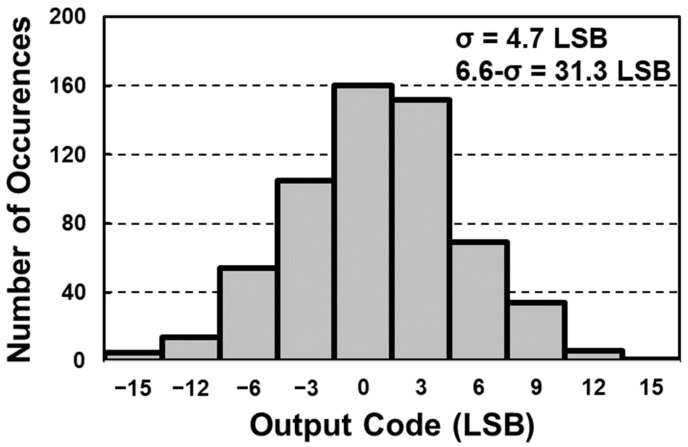
Post-layout simulation result of digitized reset data.

**Figure 11:**
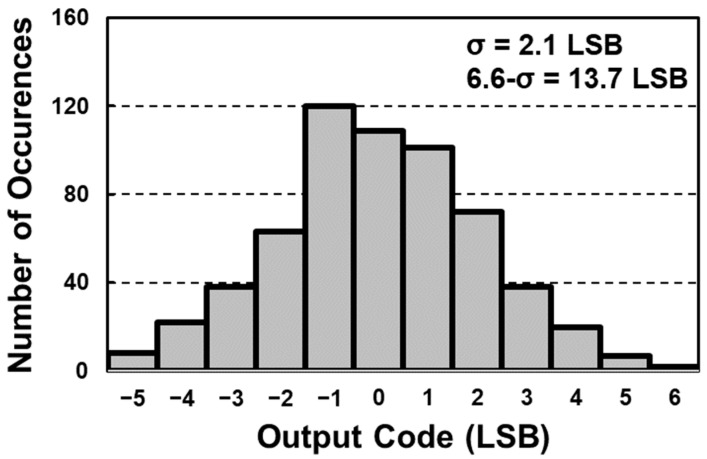
Post-layout simulation result of digitized data after digital CDS.

Figure 10 shows the histogram of the digitized reset data before applying the digital CDS technique. It shows an output distribution (1-σ) of 20 LSB, which corresponds to a 6.6-σ reliability of 120 LSB. Since the reference DAC offset is included in the digitized data without the digital CDS technique, the *x*-axis origin of the figure was moved to zero for clarity. After applying the digital CDS technique, the digitized output histogram with a 1-σ distribution of 5 LSB is achieved, as shown in Figure 11. Furthermore, thanks to the digital CDS technique, the DC offset of the histogram is also suppressed, from 0.64 LSB to 0.003 LSB.

**Figure 12:**
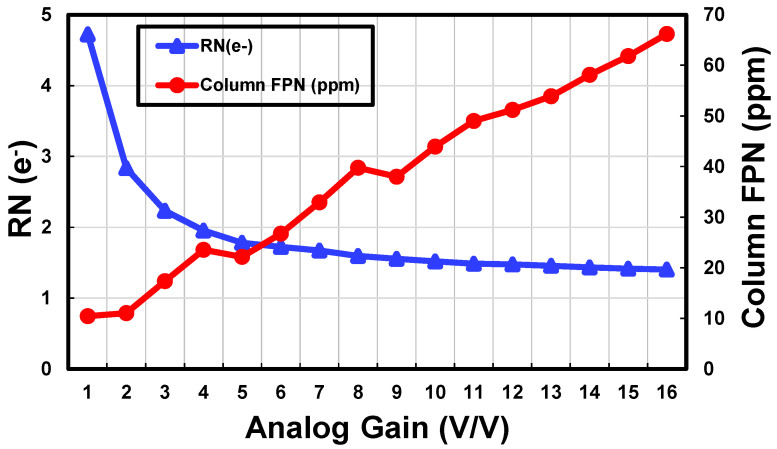
Measured random noise and column FPN versus analog gain [7].

**Figure 13:**
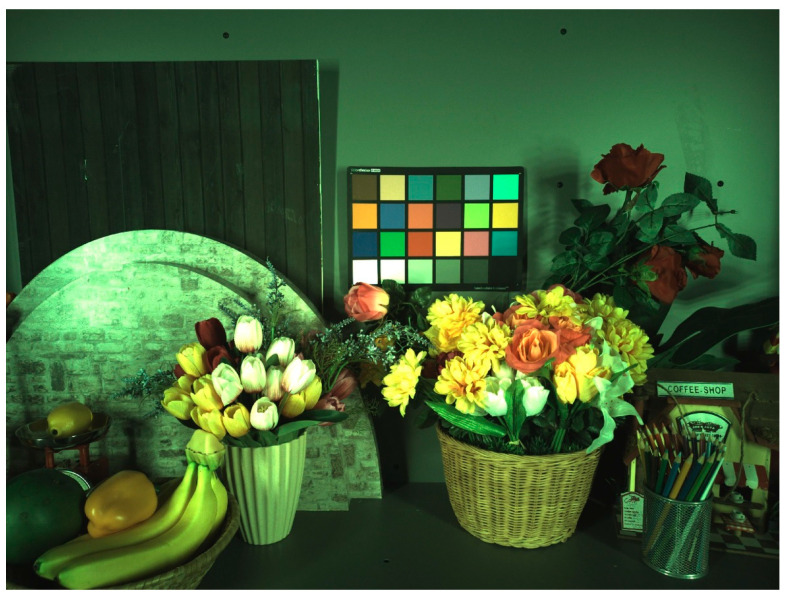
Captured image at 20 lux.

The digitizer array chip is connected to a 0.7 μm 108 MP pixel array chip in a 3-D stacked configuration for its performance verification [7]. Figure 12 shows the measured random noise (RN) and column FPN. The sample image captured by the 3-D stacked CIS at 20 lux and 10 fps is shown in Figure 13. With a one-row read-out time of 6.9 μs, an amplifier bandwidth of 1.1 MHz, and a reference DAC offset of 512 LSB, an RN of 1.4 e^-^rms and a column FPN of 66 ppm are measured at an analog gain of 16. The 108 MP imager consumes only 551 mW and also achieves a remarkable figure-of-merit (FoM) of 0.71 e^-^·nJ based on the common FoM equation for image sensor applications [10]. In Table 3, the performance of the 108 MP imager is summarized and compared with previously published works [5,11,12,14,16]. Compared to other image sensors, this work shows a remarkable FoM with a low RN.

## 5. Conclusions

This work presents a timing optimization methodology for power-efficient high-resolution image sensors with column-parallel single-slope ADCs. By optimizing the ramp reference offset and amplifier bandwidth, the power consumption in the amplifier array is reduced without compromising performance. The methodology has been successfully applied to a 108 MP 3-D stacked CMOS image sensor, resulting in a random noise of 1.4 e^-^rms, column fixed-pattern noise of 66 ppm, and FoM of 0.71 e^-^·nJ. The importance of this work lies in its ability to enhance energy efficiency in high-resolution image sensors, which allows for higher frame rates and improved overall system performance. The proposed design methodology is versatile and could be adapted for a wide range of imaging applications that demand optimized performance and reduced power consumption.

## Figures and Tables

**Table 1:** Design parameters for timing optimization examples.

Design Parameter	Value
ADC Resolution (N)	12-bit
Pixel resolution	12,000 × 9000
Frame rate	15 fps
One-row read-out time	7.4 μs
Full-scale range (*FSR*_ADC_)	800 mV
Maximum analog gain	16 V/V
Counter clock (1/*t*_CCLK_)	1 GHz
Reset-on (*T*_A_)	300 ns
Δ*RG*_OFF_	400 mV
*g*_m,SF_	60 μS
*R*_SEL_	15 kΩ
*R*_LINE_	30 kΩ
*C*_LINE_	2 pF
*I*_LOAD_	2.5 μA
*E*_TARG,B_	5 LSB
*E*_TARG,D_ = *E*_TARG,H_	0.05 LSB
Timing margin (*T*_C_ = *T*_F_)	50 ns
Settling for *OFF*_RAMP_	4*τ*_OTA1_
Dark counting (*T*_E_)	200 LSB
TG-on (*T*_G_)	500 ns
Δ*TG*_OFF_	400 mV
*COUNT*_MARGIN_	256 LSB

**Table 2:** Time budget results of the example.

Period	Time Budget
A	300 ns
B	1428 ns
C	50 ns
D	824 ns
(D’)	480 ns
E	200 ns
F	50 ns
G	500 ns
H	1392 ns
(H*)	912 ns
(H′)	480 ns
I	1256 ns
Sum (one-row)	7400 ns (=7.4 μs)

**Table 3:** Performance Summary and Comparison.

Parameter	This Work	[5]	[11]	[13]	[14]	[16]
Pixel pitch	0.7 μm	1.5 μm	2.45 μm	1.1 μm	1.1 μm	2.7 μm
# of pixels	108 MP	246 MP	133 MP	13.5 MP	33.8 MP	1.38 MP
ADC pitch/column	1.005 μm	–	–	–	4.4 μm	–
ADC height	1800 μm	–	–	–	920 μm	–
Frame rate	10 fps	5 fps	60 fps	34 fps	240 fps	120 fps
RN	1.4 e^-^_rms_	7.1 e^-^_rms_	7.7 e^-^_rms_	1.8 e^-^_rms_	3.6 e^-^_rms_	3.5 e^-^_rms_
HN	0.03 e^-^_rms_	–	–	–	–	–
Column FPN	66 ppm	–	–	–	–	–
Power Consumption	551 mW	1970 mW	11,000 mW	258 mW	3000 mW	205 mW
FoM ^1^	0.71 e^-^∙nJ	11.36 e^-^∙nJ	10.61 e^-^∙nJ	1.01 e^-^∙nJ	1.36 e^-^∙nJ	4.33 e^-^∙nJ

^1^ FoM (e^-^·nJ) = (Power × Noise)/(# of Pixels × Frame Rate).

## Data Availability

The data used in this paper can be obtained by contacting the first author.
