# Correction: Jun, J. A Comprehensive Methodology for Optimizing Read-Out Timing and Reference DAC Offset in High Frame Rate Image Sensing Systems. *Sensors* 2023, *23*, 7048

**DOI:** 10.3390/s23208432

**Published:** 2023-10-13

**Authors:** Jaehoon Jun

**Affiliations:** Department of Electrical Engineering, Inha University, Incheon 22212, Republic of Korea; jaehoon.jun@inha.ac.kr

In the original paper [1], an optimization example was presented in Section 3.7. However, due to the unrealizable condition in Table 1, there were some numerical errors in the results of the example. So, Table 1 has been updated to be more realistic (with 12-bit ADC, 4K, and 23 fps). Based on the updated Table 1, the numbers in Figure 8 and Table 2 have been corrected, and the numbers in the descriptions of the Figure and Table have also been updated. The corrected Figure 8, Table 1 and Table 2 appear below.

**Figure 8:**
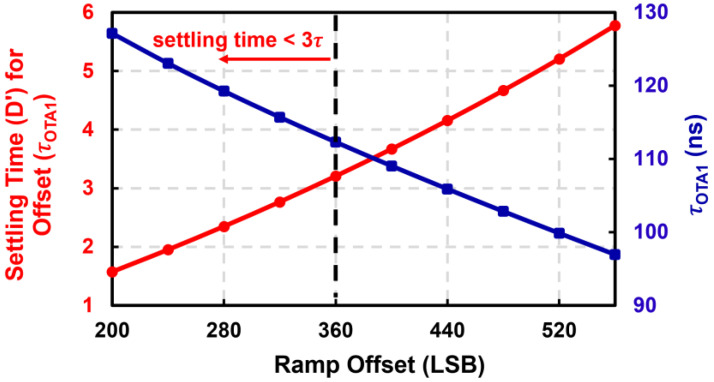
Estimated settling time for ramp offset and the time constant of the amplifier versus ramp offset.

**Table 1:** Design parameters for timing optimization examples.

Design Parameter	Value
Full-scale range (*FSR*_ADC_)	1 V
Maximum analog gain	16 V/V
Counter clock (1/*t*_CCLK_)	1 GHz
Reset-on (*T*_A_)	1 μs
Δ*RG*_OFF_	1 V
τ_SF_	0.05 μs
*I*_LOAD_	5 μA
*E*_TARG,B_	5 LSB
*E*_TARG,D_ = *E*_TARG,H_	0.05 LSB
Timing margin (*T*_C_ = *T*_F_)	50 ns
Settling for *OFF*_RAMP_	3τ_OTA1_
Dark counting (*T*_E_)	200 LSB
TG-on (*T*_G_)	1 μs
Δ*TG*_OFF_	1 V
*COUNT*_MARGIN_	256 LSB

**Table 2:** Time budget results of the example.

Period	Time Budget
A	1000 ns
B	851 ns
C	50 ns
D	885 ns
(D’)	360 ns
E	200 ns
F	50 ns
G	1000 ns
H	1635 ns
(H*)	1275 ns
(H’)	360 ns
I	4328 ns
Sum (one-row)	10,000 ns (=10 μs)

A correction has been made to the Section 3.7, Paragraphs 2 and 3, and should read:

For example, consider a 7680 × 4320 pixel array (4K) that needs to be digitized with a 12-bit ADC array at 23 fps. The ADC array needs to process the pixel output of 4320 rows 23 times in 1 s, and a one-row read-out time is then 10 μs. With a specific pixel structure, system architecture design, and circuit simulation results, design parameters for a high-resolution image sensor can be achieved, as shown in Table 1. With the design parameters, the settling time for ramp offset and the time constant of the amplifier versus ramp offset can be calculated, as shown in Figure 8. A large ramp offset is required to ensure a sufficient ramp offset settling time, which in turn requires a small time constant, which increases power consumption.

Through the iterative calculation based on the other parameters in Table 1 and the equations in Section 3, optimized time budget results can be achieved, as shown in Table 2. With the proposed timing optimization methodology, an optimal reference offset of 360 LSB was achieved. Furthermore, an optimal amplifier time constant of 112.3 ns is also derived, which is equivalent to a bandwidth of 1.42 MHz. Without optimizing the reference offset as proposed in this paper, the power efficiency of an image sensing system becomes very poor. For example, an amplifier bandwidth of 2.12 MHz would be required to maintain the same CL error with an unoptimized reference offset of 240 LSB.

In the Abstract and Section 4, minor text edits have been made to replace some unnecessary details about the previous work, with a reference for clarification.

The corrected part in the Abstract, except for the first five sentences, is as follows:

This timing optimization methodology enhances energy efficiency in high-resolution image sensors, enabling higher frame rates and improved system performance. It could be adapted for various imaging applications requiring optimized performance and reduced power consumption, making it a valuable tool for designers aiming to achieve optimal performance in power-sensitive applications.

The corrected first paragraph in Section 4 is as follows:

A power-efficient digitizer array for verifying the proposed time budgeting method is implemented in a 28-nanometer process. The prototype digitizer is designed with an optimal reference ramp offset and a 10-bit column-parallel single-slope ADC array. Figure 9 shows an annotated microphotograph of the digitizer chip, which can be stacked with a pixel chip. The comparator array and counter array are operated with a supply voltage of 2.8 V and 1 V, respectively. The peripheral blocks include a DAC for reference ramp signal generation, a voltage doubler for the pixel chip, and reference current generation.

The corrected second paragraph and Table 3 in Section 4 are as follows:

The digitizer array chip is connected to a 0.7 μm 108 MP pixel array chip in a 3-D stacked configuration for its performance verification, and the low-frequency noise is suppressed using the digital CDS technique [7]. Figure 10 shows the measured random noise (RN) and column FPN. The sample image captured using the 3-D stacked CIS at 20 lux and 10 fps is shown in Figure 11. An RN of 1.4 e^−^rms and a column FPN of 66 ppm are measured at an analog gain of 16. The 108 MP imager consumes only 551 mW and also achieves a remarkable figure-of-merit (FoM) of 0.71 e^−^·nJ based on the common FoM equation for image sensor applications [10]. In Table 3, the performance of the 108 MP imager is summarized and compared with previously published works [5,11,12,14,16]. Compared to other image sensors, this work shows a remarkable FoM with a low RN.

The corrected Figure 9 and Table 3 appear below.

**Figure 9:**
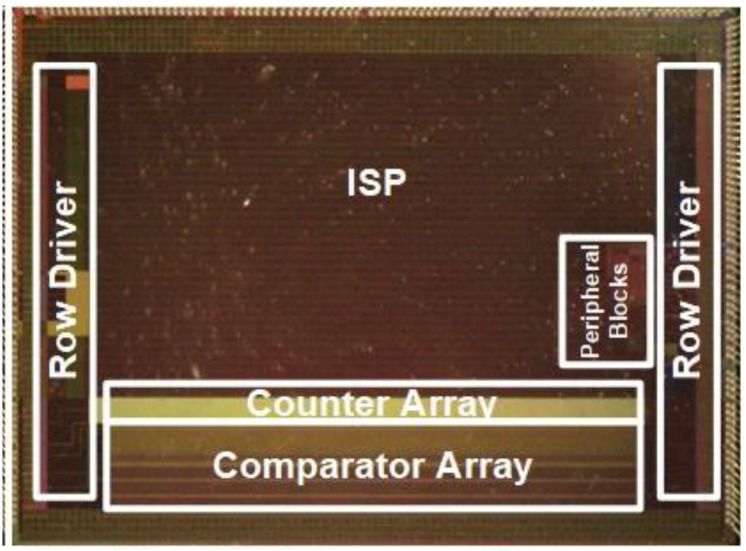
Microphotograph of the digitizer chip [7].

**Table 3:** Performance summary and comparison.

Parameter	This Work [7]	[5]	[11]	[13]	[14]	[16]
Pixel pitch	0.7 μm	1.5 μm	2.45 μm	1.1 μm	1.1 μm	2.7 μm
# of pixels	108 MP	246 MP	133 MP	13.5 MP	33.8 MP	1.38 MP
Frame rate	10 fps	5 fps	60 fps	34 fps	240 fps	120 fps
RN	1.4 e^−^_rms_	7.1 e^−^_rms_	7.7 e^−^_rms_	1.8 e^−^_rms_	3.6 e^−^_rms_	3.5 e^−^_rms_
HN	0.03 e^−^_rms_	–	–	–	–	–
Column FPN	66 ppm	–	–	–	–	–
Power Consumption	551 mW	1970 mW	11,000 mW	258 mW	3000 mW	205 mW
FoM ^1^	0.71 e^−^∙nJ	11.36 e^−^∙nJ	10.61 e^−^∙nJ	1.01 e^−^∙nJ	1.36 e^−^∙nJ	4.33 e^−^∙nJ

^1^ FoM (e^−^∙nJ) = (Power × Noise)/(# of Pixels × Frame Rate).

The author apologizes for any inconvenience caused and states that the scientific conclusions are unaffected. This correction was approved by the Academic Editor. The original publication has been updated.
